# A case of esophageal granular cell tumor diagnosed by mucosal incision-assisted biopsy

**DOI:** 10.1007/s12328-021-01535-y

**Published:** 2021-10-22

**Authors:** Yasuhiro Inokuchi, Mamoru Watanabe, Kei Hayashi, Yoshihiro Kaneta, Mitsuhiro Furuta, Nozomu Machida, Shin Maeda

**Affiliations:** 1 Department of Gastroenterology, Kanagawa Cancer Center, 2-3-2 Nakao, Asahi-ku, Yokohama, Kanagawa 241-8515 Japan; 2 Department of Gastroenterology, Yokohama City University, 3-9 Fukuura, Kanazawa-ku, Yokohama, Kanagawa, 236-0004 Japan

**Keywords:** Mucosal incision-assisted biopsy (MIAB), Granular cell tumor (GCT), Endoscopic ultrasound-fine needle aspiration (EUS-FNA), Endoscopic submucosal dissection (ESD), Positron emission tomography-computed tomography (PET-CT)

## Abstract

For an esophageal submucosal mass suspicious of granular cell tumor (GCT) based on gross appearance and endoscopic ultrasound findings, a sufficient number of biopsy specimens is required for a definite diagnosis using immunohistochemical examination. When the specimen obtained by forceps biopsy is insufficient, endoscopic ultrasound-fine needle aspiration (EUS-FNA) is believed to be an useful alternative. However, it may be difficult to obtain an adequate amount of tumor material using EUS-FNA. Mucosal incision-assisted biopsy (MIAB) is a simple method that can collect larger amounts of specimens. This procedure is helpful for physicians who encounter the problem of obtaining an adequate amount of biopsy material from esophageal tumors suspicious for GCT. We present a case of esophageal GCT that was successfully diagnosed through MIAB.

## Introduction

A granular cell tumor is a tumor which can develop in various organs, such as skin, oral cavity, lung, bronchi, bladder, uterus and gastrointestinal tract. Ultrasonography-guided fine needle aspiration is useful in the diagnosis of granular cell tumors (GCT) in breast [1, 2]. Additionally, in certain conditions when the usual biopsy by forceps fails to confirm a definite diagnosis of esophageal GCT, it is believed that endoscopic ultrasound-fine needle aspiration (EUS-FNA) is a useful alternative. A few reports have indicated that minute specimens obtained via EUS-FNA provided a definite diagnosis of gastrointestinal GCT [3]. However, in most previous studies, a definite diagnosis of GCT was made using the resected tumor itself after endoscopic or surgical resection [4–8]. John et al. reported the utility of EUS-FNA, but the final diagnosis of GCT was made using specimens obtained by a computed tomography (CT)-guided core needle biopsy [9]. In this study, we introduced a mucosal incision-assisted biopsy (MIAB) for GCT; this is a simple biopsy method to obtain a sufficient number of tumor specimens for a histopathological diagnosis of GCT.

## Case report

**Fig. 1:**
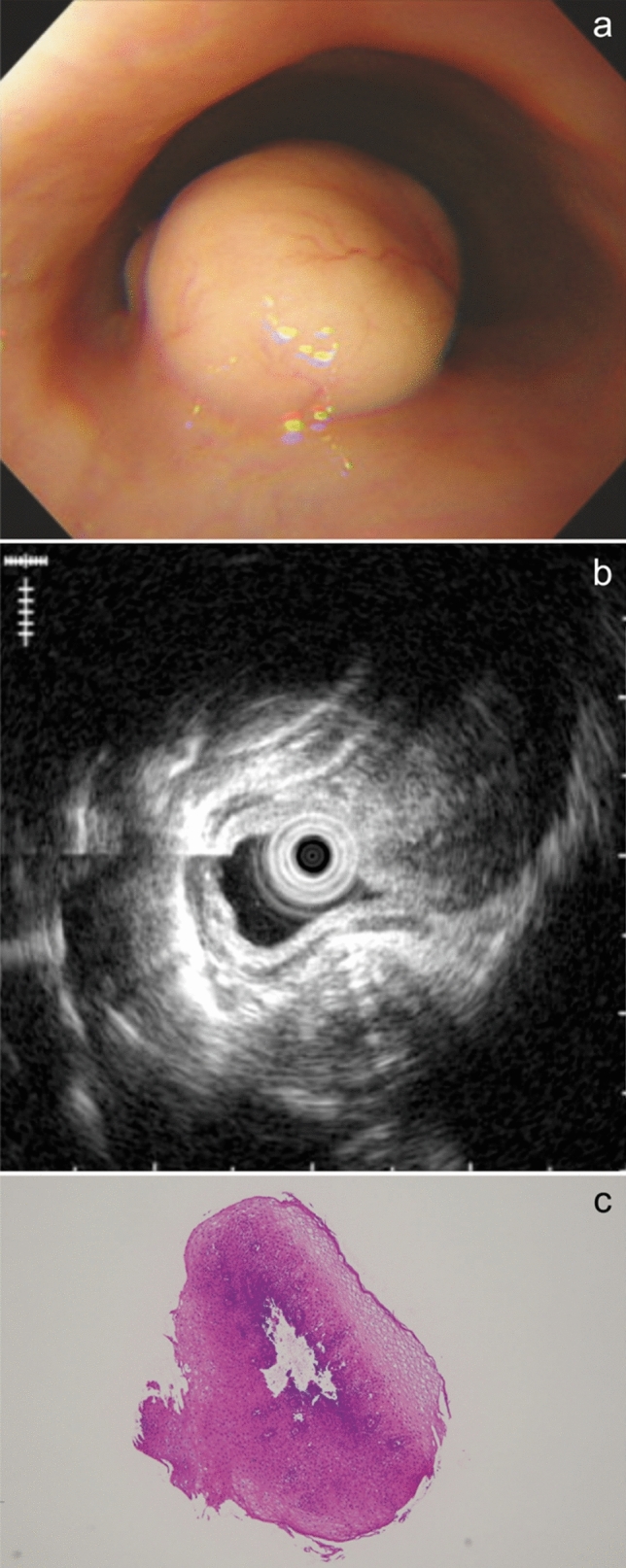
Endoscopic appearance of the esophageal tumor. **a** A yellowish tumor covered by the esophageal epithelium with dilated vessels. **b** A grayscale homogenous tumor was seen in the submucosal layer in EUS findings, by thin probe of 20 MHz. **c** Biopsy specimens taken by forceps contained no tumor cells in H-E staining. *EUS* esophageal ultrasound; *H-E* Hematoxylin–Eosin

**Fig. 2:**
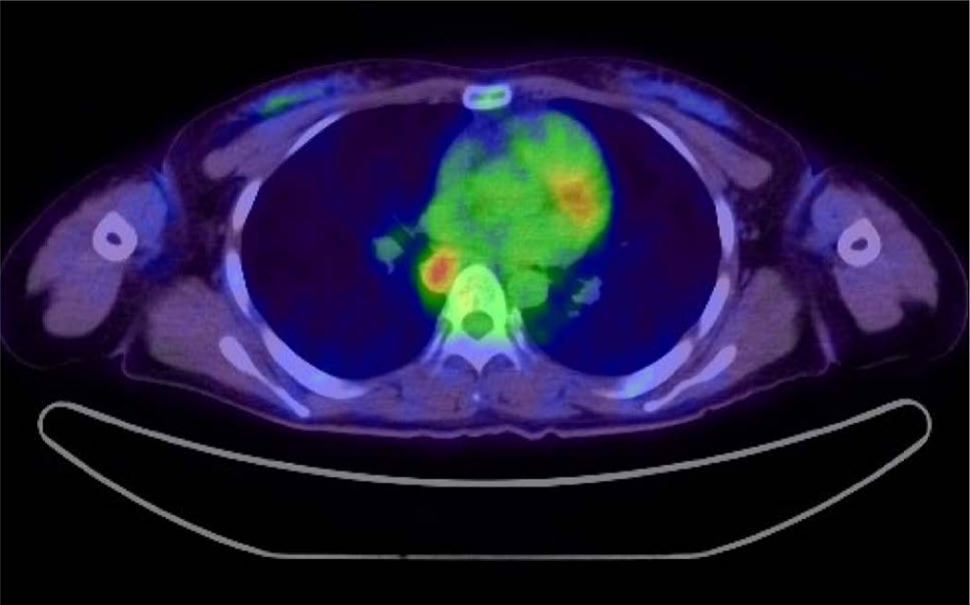
Enhanced uptake of FDG into the esophageal tumor was detected in PET-CT. *FDG* 2-deoxy-2-fluoro-18F-D-glucopyranose; *PET-CT* Positron emission tomography-computed tomography

A 33-year-old female patient who had been diagnosed with non-Hodgkin diffuse large B-cell lymphoma underwent screening esophagogastroduodenoscopy for staging before treatment. A tumor of non-epithelial origin was discovered at the posterior wall of the middle intrathoracic esophagus. On gross examination, it was a yellowish submucosal mass with a size of 3 × 1.5 cm, covered by normal mucosa (Fig. 1a). EUS suggested a submucosal limitation without invasion of the muscularis propria, however that could not be confirmed due to the heart beat and deep attenuation of the echo (Fig. 1b). Transmucosal forceps biopsy was repeated over two different days, but the obtained specimens were not sufficient for histopathological diagnosis of the tumor, though a boring biopsy was performed at the second opportunity (Fig. 1c). Positron emission tomography-computed tomography (PET-CT) scan revealed an uptake of 2-deoxy-2-fluoro-18F-D-glucopyranose (FDG) in the esophageal tumor (Fig. 2). The tumor had a possibility of being a lymphomatous lesion, and because the presence of an esophageal lymphomatous lesion might affect the staging, MIAB was performed.

**Fig. 3:**
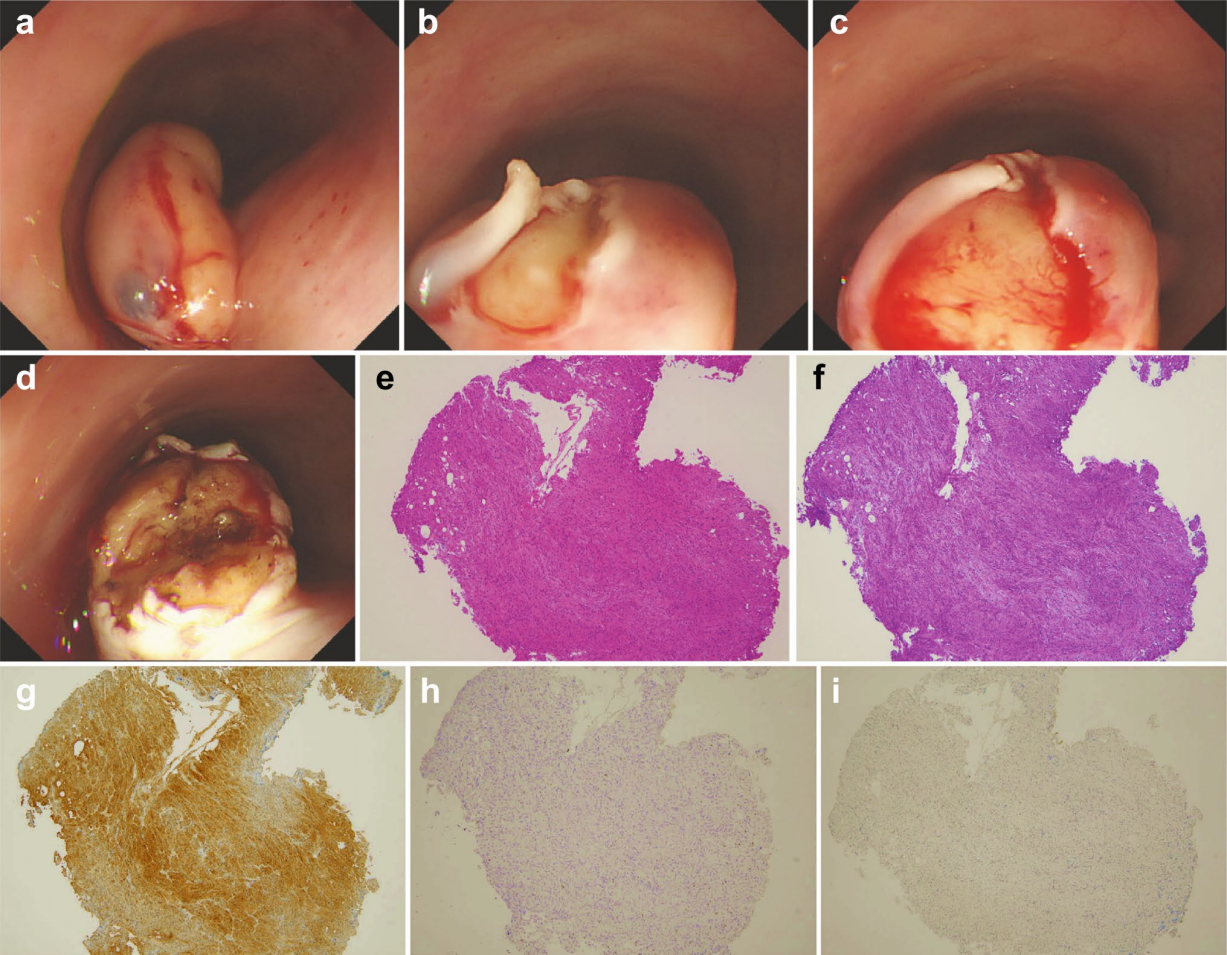
Mucosal incision-assisted biopsy procedure. **a** Submucosal injection with normal saline. (**b**, **c**) Longitudinal mucosal incision using DualKnife. **d** Biopsy of the tumor under direct vision. **e** H-E stain of the biopsy specimens showed small tumor cells with eosinophilic cytoplasm. **f** PAS stain revealed cytoplasm of tumor cells were rich with PAS-positive granules. **g** Immunohistochemical staining for S-100 protein showed tumor cells were positive for S-100 protein. **h** Immunohistochemical staining for KIT protein showed tumor cells were negative for KIT. **i** Immunohistochemical staining for Desmin showed tumor cells were negative for Desmin. *H-E* Hematoxylin–Eosin; *PAS* periodic acid Schiff

Normal saline was injected into the submucosal layer over the tumor (Fig. 3a). Then, the mucosa covering the tumor was incised longitudinally using DualKnife (Olympus Optical Co., Ltd., Tokyo, Japan) to expose the lesion (Fig. 3b, c). Forceps biopsy was performed using disposable biopsy forceps with a needle in the center of the cup (FB-240U, Olympus Optical Co., Ltd., Tokyo, Japan) under direct visualization of the tumor (Fig. 3d). Finally, the tumor was diagnosed as GCT on pathological assessment including immunohistochemical examination, as the tumor was positive for S-100 and negative for both KIT and Desmin (Fig. 3e–i).

**Fig. 4:**
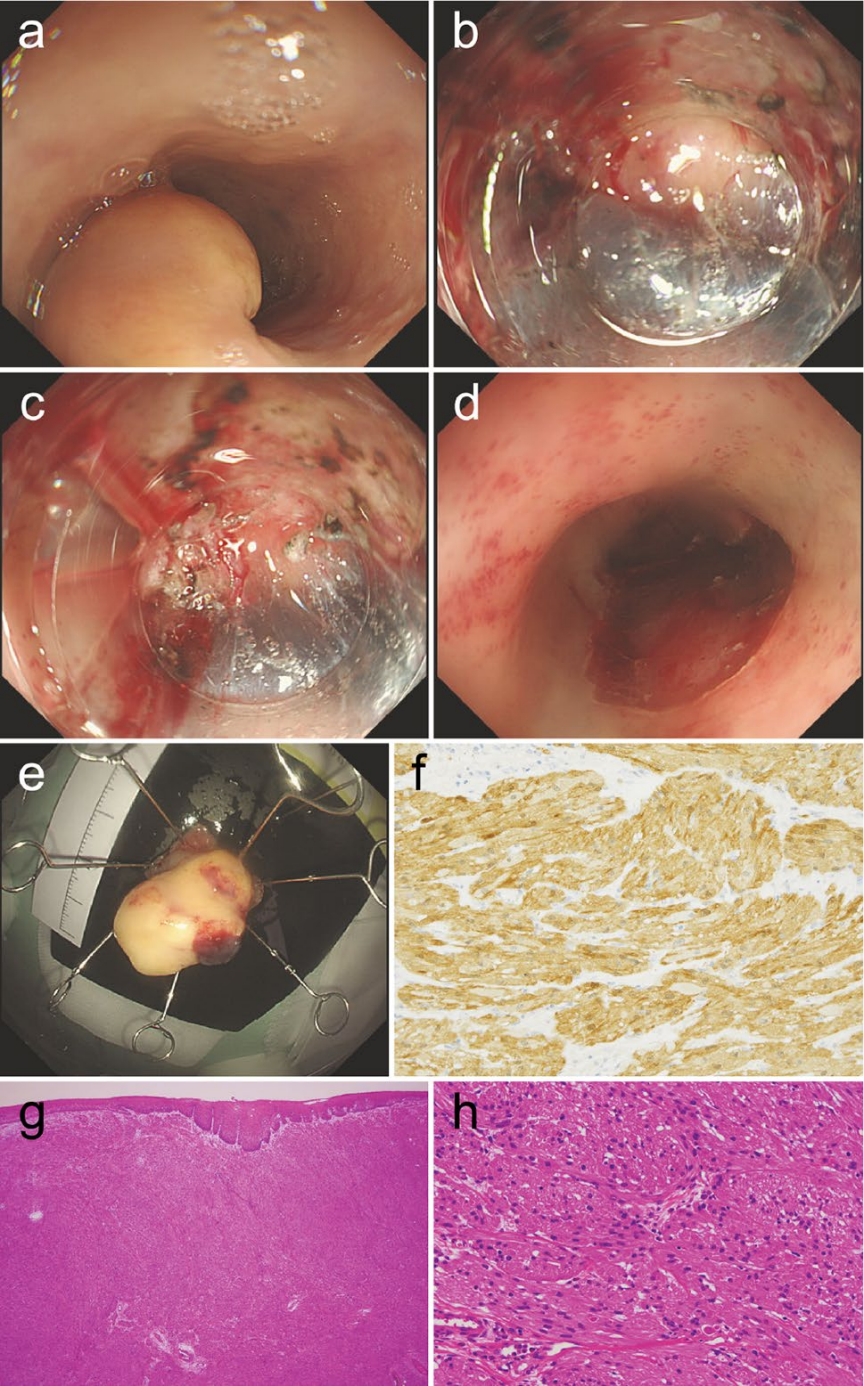
**a** A yellowish submucosal tumor at the middle intrathoracic esophagus. The tumor was completely dissected without any remnant lesion. (**b**, **c**) The tumor was resected by ESD under direct vision from the deeper side using a scissor-type device. **d** The wound from ESD showed no damage to the muscular layer. **e** Resected yellowish tumor. **f** Immunohistochemical staining for S-100 protein showed the tumor cells were positive for S-100. **g** H-E stain (× 40) of resected tumor showed tumor cells with eosinophilic cytoplasm proliferating in submucosa in solid alveolar form. **h** Under high magnification (× 400), tumor cells appeared as small cells with small uniform nuclei and eosinophilic and granule-rich cytoplasm. *ESD* Endoscopic submucosal dissection; *H-E* Hematoxylin–Eosin

Two months after the diagnosis, endoscopic submucosal dissection (ESD) was performed as the usual treatment for esophageal GCT [10]. Care was taken to avoid burning the tumor surface, and SB knife Jr. (Sumitomo Bakelite, Tokyo, Japan), which is a scissor-type device, was used in dissecting the deep submucosal layer. The tumor was easily and safely resected under direct vision in 35 min (Fig. 4a–e). Macroscopically, the tumor was 21 mm in size, and its cut surface was yellowish. Histopathological assessment showed small cells with small uniform nuclei and eosinophilic and granule-rich cytoplasm. These cells proliferated in solid alveolar form. Immunohistologically, the tumor cells were positive for S-100 protein (Fig. 4f–h). On the other hand, they were negative for SMA, Desmin, and CD34. There was no division of the cells or necrosis, and Ki-67 index was less than 1%.

**Fig. 5:**
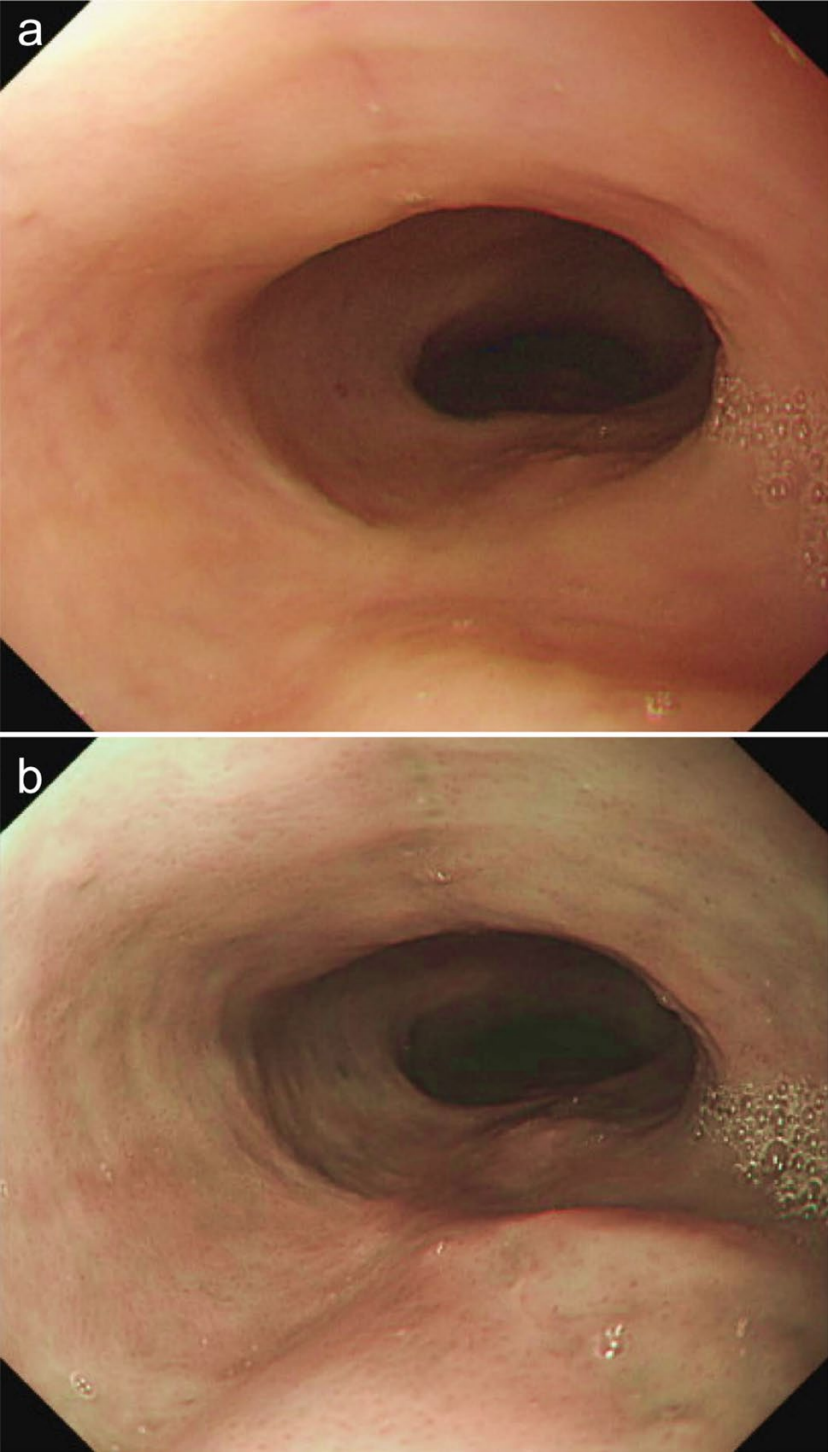
Post-ESD scar at six months after ESD. **a** White light imaging. **b** Narrow band imaging. *ESD* Endoscopic submucosal dissection

The post-ESD ulcer was completely healed with scar formation on the follow-up endoscopy performed six months after ESD (Fig. 5a, b). No recurrence was seen within three years after ESD.

## Discussion

We encountered an esophageal submucosal tumor with increased FDG uptake in PET-CT and a low echoic nature in EUS, which could not be diagnosed after two forceps biopsies. As the patient had been previously diagnosed with non-Hodgkin diffuse large B-cell lymphoma, we initially supposed the esophageal tumor was a lymphoma lesion. If such was the case, the staging of the malignant lymphoma might be affected, and furthermore, it could be treated along with other lesions by general chemotherapy. However, there were other possibilities in this case [11]. If it was a malignant submucosal tumor such as gastrointestinal stromal tumor (GIST) and leiomyosarcoma which might also present with low echoic aspect in EUS and increased FDG uptake in PET-CT [12, 13], it must be resected surgically apart from lymphoma. We also supposed the tumor to be GCT, which was possible to remove endoscopically, because it was yellowish and low echoic, even if it was atypical due to the increased FDG uptake. There were also a few reports that showed increased FDG uptake in benign leiomyoma, which is the most common submucosal tumor that develops in the esophagus, and it is unnecessary to remove it if no symptoms exist [14, 15]. Therefore, we had to confirm the definite diagnosis of esophageal submucosal tumor to decide a plan of treatment.

Gross appearance and EUS images are useful in predicting esophageal tumors as GCTs. A typical aspect of an esophageal GCT is a grayish-white or yellowish protuberant lesion covered by normal esophageal epithelium. The top of the lesion is frequently depressed, and its appearance is described as molar-like [8]. EUS is also valuable in distinguishing GCTs from other submucosal lesions. In EUS images, GCTs generally appear as low echoic lesions restricted within the submucosal layer, with average grayscale values greater than those of the muscularis propria [5]. Nevertheless, in the definite diagnosis of GCT, histopathological diagnosis including immunohistochemical staining is indispensable. Microscopically, the tumor is comprised of small cells with small uniform nuclei and granule-rich cytoplasm positive for S-100 protein. Unlike GIST or leiomyoma, GCT is negative for KIT protein and Desmin.

As most esophageal GCTs are benign [4, 16], Voskull et al. recommended performing follow-up endoscopy once a year after the initial diagnosis for esophageal GCT patients without rapid growth until dysphagia occurs [17]. Nonetheless, in confirmed cases of esophageal GCT, the possibility of malignancy must be considered. The assessment of malignant potential based on size and cellular pleomorphism is not reliable [1]. Although a few reports indicated the usefulness of a PET-CT scan in distinguishing malignant GCTs from benign ones, it is still unclear whether they are indeed distinguishable by PET-CT. Moreover, there is currently no standard cutoff value of FDG uptake [7, 18].

Stašek et al. recommended that GCT, which might have potentially malignant features, should be removed early on, as the prognosis of malignant cases is poor [7]. Accordingly, the timely diagnosis and resection of GCTs is critical. We recommend performing MIAB for lesions suspected of GCT based on gross appearance and EUS findings which are not successfully diagnosed by forceps biopsy. MIAB is superior to EUS-FNA because of the following advantages: (1) a greater amount of specimen is collected, (2) the capability to perform coagulation hemostasis when bleeding occurs, (3) relatively less technique sensitivity, and (4) the absence of the need for a specific endoscope. On the other hand, a disadvantage of MIAB is that the procedure is not covered by insurance in Japan. It is dealt likewise standard forceps biopsy in point of charge, therefore the cost of devices used for MIAB are included in amount for standard forceps biopsy paid by patients and health insurance society. In the present case, we used DualKnife which is a disposable knife for mucosal cutting. To reduce the cost, a reusable needle-type knife may be considered. Once GCT is diagnosed, endoscopic resection, especially ESD, is recommended as an approach for total resection satisfying negative horizontal and vertical margins, in cases where esophageal GCT is revealed to be limited in the submucosal layer by EUS.
